# Correlation Analysis Between Multi-Drug Resistance Phenotype and Virulence Factor Expression of Clinical *Pseudomonas aeruginosa*

**DOI:** 10.3390/cimb47010050

**Published:** 2025-01-15

**Authors:** Wenli Xu, Runcheng Zhou, Jingwei Pan, Zhuangcong Liu, Xuyu Huang, Yueqiao Lin, Nan Li, Kecan Chen, Wenbo Sun, Yi Deng, Anping Yang, Xin Chen

**Affiliations:** School of Medicine, Foshan University, Foshan 528225, Chinaliuzhuangcong400@163.com (Z.L.);

**Keywords:** *Pseudomonas aeruginosa*, multi-drug resistance, virulence factor, correlation analysis, clinical separation

## Abstract

*Pseudomonas aeruginosa* (PA), as a common pathogen of nosocomial infections, has been experiencing an increasing rate of drug resistance with the widespread use and abuse of antimicrobial drugs. High-drug-resistance and high-virulence phenotypes are two distinctive features of the strong pathogenicity of multi-drug-resistant PA. Exploring the characterization of virulence factor expression and its relationship with the multi-drug resistance phenotype is essential to reduce the further development of resistance as well as a high standard of infection prevention and control. A total of 50 PA isolated from clinical practice were collected. The *Kirby-Bauer*  test was used for drug-sensitive screening, and the results showed that 16 strains were resistant and 16 strains were sensitive. The drug resistance rate of multi-drug-resistant PA against cefepime, cefazolin, ampicillin, and imipenem was up to 100%. The multi-drug-resistant groups were superior in producing pyocyanin and forming biofilm to the sensitive groups. The distribution of isolates with different swarming motility capacities and elastase levels did not show pronounced differences among the multi-drug-resistant and sensitive groups. In addition, biofilm formation was moderately associated with imipenem resistance. Among the strains with strong virulence factor expression, the gene bands showed little difference, suggesting that the gene is highly homologous. The virulence factor matrix analysis showed that there were different degrees of correlation among the 4 virulence factors. The correlation between multidrug-resistant PA and virulence factor expression is complex. PA, which were good at producing pyocyain and forming biofilm, were highly resistant to cephalosporins, beta-lactams and carbepenems; hence, such drugs are not proper for anti-infective treatment in clinics.

## 1. Introduction

*Pseudomonas aeruginosa* (PA) is an opportunistic pathogen that involves infections in many parts of the body, including the respiratory system, urinary system, skin, and soft tissues, especially in patients with cystic fibrosis (CF) and pulmonary infections. As a common pathogen causing nosocomial infections, the resistance rate of PA continues to increase with the widespread use and abuse of antibiotics [1,2]. The hospital infection situation in PA is becoming increasingly severe, with drug-resistant strains constantly emerging and mostly exhibiting multi-drug resistance (MDR) [3]. Multi-drug-resistant *Pseudomonas aeruginosa* (MDR-PA) is often associated with global epidemic outbreaks, resulting in high incidence rate and mortality, which pose a huge challenge for clinical treatment of MDR-PA infection [4,5].

High drug resistance and high virulence phenotype are two significant characteristics of the strong pathogenicity of MDR-PA. With the increase of high drug resistance and infection rates, empirical medication used by clinical doctors is gradually becoming less applicable. According to reports, the monitoring and control of important epidemiological pathogens have revealed that 35.8%, 36.8%, and 36.6% of PA are resistant to meropenem, imipenem, and cefotaxime, respectively [6,7]. Therefore, exploring the characteristics of virulence factor expression and its relationship with multi-drug resistance phenotypes is crucial for reducing the further development of drug resistance and achieving high standards of infection prevention and control.

In this work, we isolated 50 strains of PA from clinical samples and screened them for multi-drug-resistant strains (resistant to three or more different types of antibiotics) and sensitive strains (sensitive to antibiotics or resistant to fewer than two types of antibiotics) using eight conventional antibiotics. Subsequently, the correlation between the PA multi-drug resistance phenotype and virulence factors such as biofilm, elastase, cluster motility, and pyocyanin was analyzed, providing new ideas and references for the clinical treatment of MDR-PA infections and the development of drug resistance reduction.

## 2. Materials and Methods

### 2.1. Materials

Glucose and yeast extract were purchased from Shenggong Bioengineering Co., Ltd. (Shanghai, China). Tryptone was purchased from Biosharp (Guangzhou, China). Agar powder was purchased from Haibo Biotechnology (Qingdao, China). Nutritional meat soup powder was purchased from Guangdong Huankai Microbial Technology Co., Ltd. (Guangzhou, China). Defatted milk powder and Oxford cup were purchased from Shanghai Sanshe Industrial Co., Ltd. (Shanghai, China). Chloroform was purchased from Guangzhou Chemical Reagent (Guangzhou, China). M hydrochloric acid, nutrient broth (NB) medium, MH broth medium, and 1% crystal violet were purchased from Zhuhai Beiso Biotechnology Co., Ltd. (Zhuhai, China), while 99% acetic acid was purchased from Guangdong Guanghua Technology Co., Ltd. (Guangzhou, China). Antibiotic susceptibility discs of cefazolin (CZ, 30 μg/piece), cefepime (CPI, 30 μg/piece), ampicillin (AMP, 10 μg/piece), imipenem (IPM, 10 μg/piece), ciprofloxacin (CIP, 5 μg/piece), norfloxacin (NOR, 10 μg/piece), gentamicin (GEN, 10 μg/piece), and piperacillin (PIP, 100 μg/piece) were purchased from Hangzhou Microbial Reagent Co., Ltd. (Hangzhou, China). DNA staining agent was purchased from Beijing Dingguo Changsheng Biotechnology Co., Ltd. (Beijing, China). TAE solution (Tris acetic acid EDTA) and PremixTaq were purchased from Shanghai Shenggong Bioengineering Technology Service Co., Ltd. (Shanghai, China). Primer M13 was synthesized by Shanghai Shenggong Bioengineering Co., Ltd. (Shanghai, China), and 50 clinical strains of PA (ATCC27853) were collected and isolated from the Clinical Laboratory Department of Meizhou People’s Hospital (Guangdong, China). PA (ATCC27853) was purchased as a quality control strain from the Clinical Inspection Center of the National Health Commission (Beijing, China).

### 2.2. Methods

#### 2.2.1. Antibiotic Sensitivity Test

The *Kirby-Bauer* method was used to screen 50 strains of PA isolated clinically. The PA bacterial suspension that had grown to the logarithmic stage was collected from the plate and was diluted to a concentration of 1.5 × 10^8^ CFU/mL in nutrient broth liquid medium, while 100 μL of bacterial suspension was taken and evenly coated onto the entire surface of the Mueller Hinton petri dish using a coating rod (Beyotime, Shanghai, China). Then, antibiotic discs such as imipenem, ciprofloxacin, gentamicin, and cefepime were placed on the agar surface. After 24 h of cultivation at 37 °C, the diameter of the inhibition zone was measured using a vernier caliper (NSCING, Nanjing, China) and recorded. The quality control strain (ATCC27853) was the control group. The inflection point of the drug susceptibility test was determined according to the 2023 CLSI standard, and sensitive PA (S-PA) and MDR-PA were preliminarily screened [8].

#### 2.2.2. Determination of PA Biofilm by Crystal Violet Staining Method

The PA bacterial suspension was diluted in nutrient broth liquid culture medium to a concentration of 1.5 × 10^8^ CFU/mL, 100 μL of that was added to each of the 5 parallel wells in a 96 well plate, and they were incubated at 37 °C for 24 h. After suctioning out the bacterial solution, the sample wells were washed three times with water. After drying, 120 μL of 0.1% crystal violet was added, and the biofilm was stained for 30 min. Then, the wells were washed until the effluent was colorless, 200 μL of 33% acetic acid was added, and the mixture was measured in an ELISA reader with a wavelength of 590 nm [9].

#### 2.2.3. Determination of Pyocyanin

The bacterial suspension was adjusted to OD_600_ = 1.5 under UV spectrophotometry. The bacterial suspension was centrifuged at 8500 rmp for 10 min; then, 5 mL of the supernatant was removed and 3 mL of chloroform was added. The mixture was shaken well and centrifuged at 8500 rmp for 5 min. After that, 2 mL of the lower chloroform phase was taken and 1.2 mL of 0.2 M hydrochloric acid was added. The mixture was shaken well and centrifuged at 8500 rmp for 5 min again, and 200 μL of the upper aqueous phase (pink) was sucked out and measured in an ELISA reader (Guangzhou Buqian Biotechnology Co., Ltd., Guangzhou, China) with a wavelength of 520 nm [10].

#### 2.2.4. Elastase Assay

The high-pressure sterilized Oxford Cup (Shanghai Sanshe Industrial Co., Ltd., Shanghai, China) was placed on the skim milk plate in the ultra-clean workbench and gently pressed it. In total, 100 μL of bacterial solution with 0.5 McPherson turbidity was injected in Oxford Cup and then cultivated at 37 °C for 24 h. The size of the dissolution ring on the skim milk plate was determined [11].

#### 2.2.5. Measurement of Cluster Movement Ability

The bacterial suspension was adjusted to OD_600_ = 1.0 under UV spectrophotometry (Shanghai Youke Instrument Co., Ltd., Shanghai, China), 1 μL of bacterial solution was gently dropped onto the center of the plate, and then the plate was incubated at 37 °C for 48 h. After that, the movement of bacterial clusters on a tablet was observed [12]. Image J version 1.54m software was used to scan and compare the area size of cluster movement of all clinical isolates.

#### 2.2.6. Homology Analysis of Strain DNA

The colonies in the plate were collected in 100 μL of water, boiled at 100 °C for 10 min, and then centrifuged at 2528 rmp for 10 min, and the supernatant was taken as a template. Preliminary typing analysis of the most virulent bacteria was conducted using random primer PCR. According to paper [13], a short random primer M13 (5′-GACGGCCAGT-3′) was synthesized. Each reaction system contained 10 μL of Taq enzyme, 2 μL of primer, 2 μL of template, and 6 μL of ddH_2_O. The reaction procedure was as follows: pre-denaturation at 94 °C for 5 min, denaturation at 94 °C for 30 s, annealing at 36 °C for 45 s, extension at 72 °C for 2 min, cycling for 40 cycles, and finally extension at 72 °C for 7 min. In total, 1.5 g of agarose powder was weighed and dissolved in 100 mL of TAE solution and heated at 100 °C for 2 min to dissolve. Then, 10 μL of GeneRed nucleic acid dye was added. After coagulation, the amplified product was added to the agarose gel. The agarose gel block electrophoresis was performed, and gel imager was used to take photos for observation [14].

#### 2.2.7. Statistical Analysis

GraphPad Prism version 9.5.0 software was used for descriptive statistical analysis. A bilateral independent sample *t*-test was performed to compare the differences in susceptibility tests, biofilm formation, growth (cluster movement), pyocyanin production, and elastase measurements between MDR and non-MDR strains of PA. The correlation matrix was calculated to evaluate the correlation between the measured values corresponding to the expression of virulence determinants. In addition, the correlation between clinical antibiotic resistance and biofilm formation was also analyzed. The PA were classified based on their sensitivity/resistance to antibiotics, and a score of 0.5/1 was obtained based on the classification of their biofilm formation ability (weak and absent/moderate). Based on the value of the Pearson correlation coefficient (*r*), the relationship between variables is determined, in which |*r*| < 0.3 indicates a weak correlation, 0.3 < |*r*| < 0.5 indicates a moderate correlation, and 0.5 < |*r*| < 0.85 indicates a strong correlation.

## 3. Results

### 3.1. Antibiotic Sensitivity Disk Method

**Figure 1:**
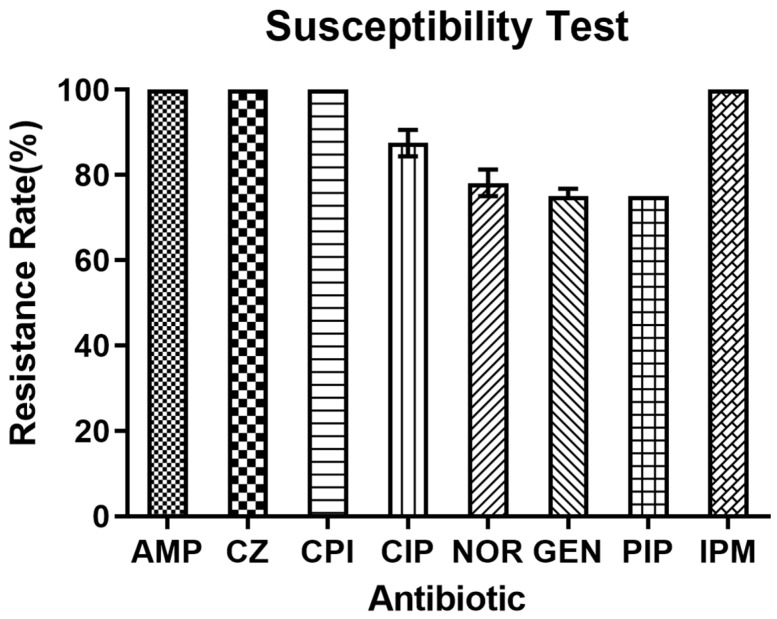
Results of antimicrobial drug screening for MDR-PA (IPM: imipenem, GEN: gentamicin, AMP: ampicillin, NOR: norfloxacin, CIP: ciprofloxacin, CZ: cefoperazone, CPI: cefotaxime, and PIP: piperacillin).

In total, 16 strains of MDR-PA and 34 strains of sensitive PA (S-PA) were identified by drug resistance screening. The resistance rates of 16 strains of MDR-PA to cefazolin, cefepime, ampicillin, and imipenem reached 100%, followed by ciprofloxacin with a resistance rate of 87.5%. The resistance rates to norfloxacin, gentamicin, and piperacillin were the lowest (Figure 1). Subsequently, 16 strains of MDR-PA and 16 strains of S-PA were selected for subsequent experiments due to the rationality of grouping.

### 3.2. Characteristics of Clinical PA Strains

**Figure 2:**
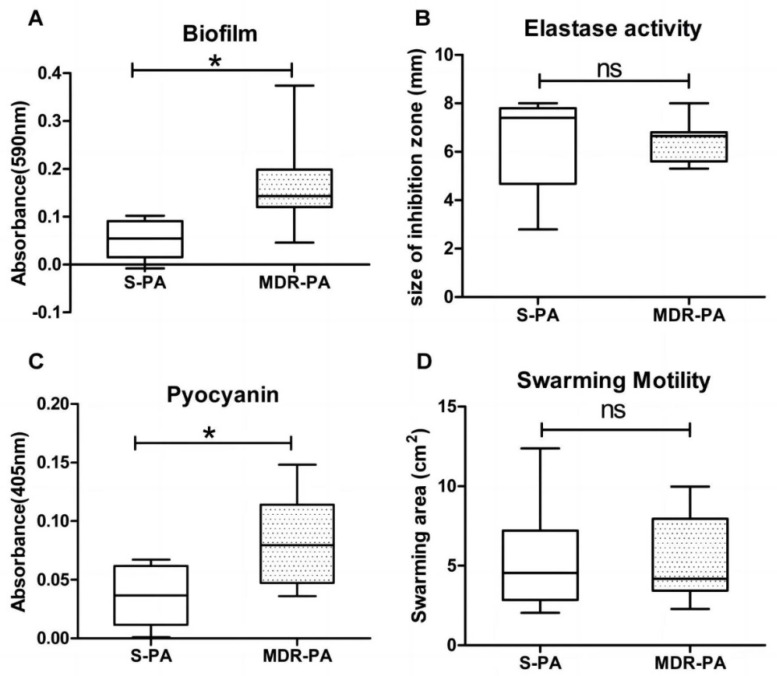
Comparison of virulence factors between S-PA and MDR-PA groups. Comparison of overall levels of biofilm formation (**A**) between S-PA group and MDR-PA group. Comparison of overall levels of elastase secretion (**B**) between S-PA group and MDR-PA group. Comparison of the overall level of collective exercise ability (**C**) between S-PA group and MDR-PA group. Comparison of overall levels of pyocyanin secretion (**D**) between S-PA group and MDR-PA group (* indicates significant difference, and ns indicates no significant difference).

Out of 32 PA strains, 30 (93.75%) had the ability to form biofilms. Among the strains with biofilm activity, 26.7% and 73.3%, respectively, had moderate and weak biofilm formation degrees (Table 1). It was determined that the amount of biofilm formation in the MDR group was significantly higher than that in the sensitive group (*p* < 0.05), as shown in Figure 2A. In addition, as shown in Table 2, this experiment also investigated the correlation between the degree of biofilm formation and the resistance of PA to eight antibiotics. The results showed that only a moderate correlation was found between biofilm formation and imipenem resistance (*r* = 0.488, *p* < 0.05), and no other significant correlations were observed.

**Figure 3:**
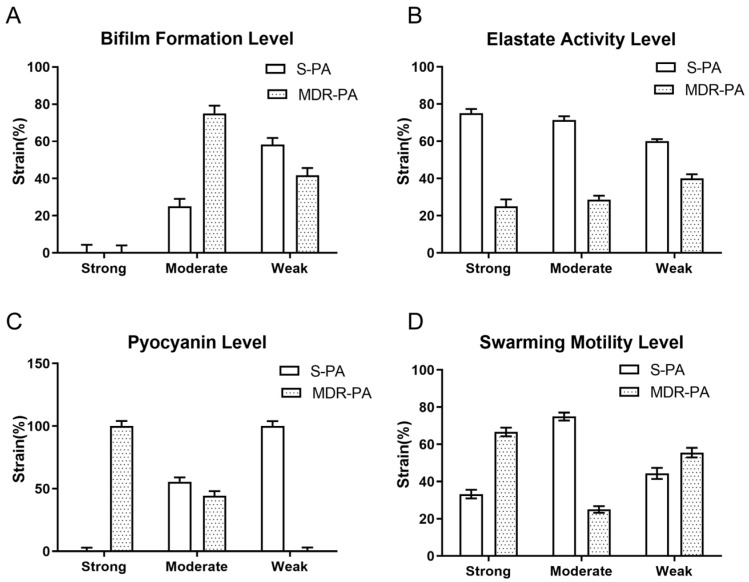
Comparison of strength classes of four virulence factors of PA. Comparison of biofilm formation strength levels (**A**) between S-PA group and MDR-PA group (negative: OD ≤ OD_c_, weakly positive: OD_c_ < OD ≤ 2OD_c_, moderately positive: 2ODc < OD ≤ 4OD_c_, and strongly positive: OD > 4OD_c_). Comparison of elastase secretion intensity levels (**B**) between S-PA group and MDR-PA group (weak: ring diameter < 5.5 mm, moderate: 5.5 mm ≤ ring diameter < 7.5 mm, and strong: ring diameter ≥ 7.5 mm). Comparison of cluster exercise ability levels (**C**) between S-PA group and MDR-PA group (weak: areas < 4 cm^2^, moderate: 4 cm^2^ ≤ areas < 8 cm^2^, and strong: areas ≥ 8 cm^2^). Comparison of secretion intensity levels of pyocyanin (**D**) between S-PA group and MDR-PA group (weak: OD_520_ < 0.020, moderate: 0.020 ≤ OD_520_ < 0.100, and strong: OD_520_ ≥ 0.100).

The distribution of strains with higher levels of elastase secretion in the S-PA group was significantly higher than that in the MDR-PA group (Figure 3B). It was determined that there was no significant difference in the average secretion level of elastase between the MDR-PA group and the S-PA group (Figure 2B).

**Figure 4:**
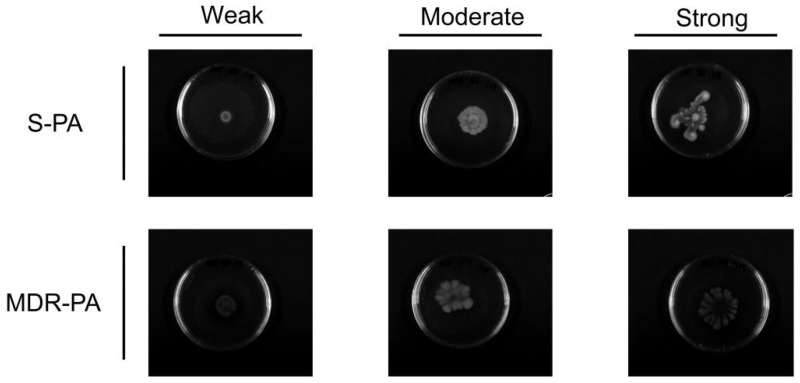
Different phenotypes formed by PA cluster movement.

All clinical isolates possessed cluster motility and formed different phenotypes (Figure 4). However, there was no significant difference in the distribution of cluster mobility between the MDR-PA group and the S-PA group (Figure 3D). After statistical analysis, it was found that there is no correlation between the strength of cluster movement ability and the level of antibiotic resistance (Figure 2D).

The 32 strains obtained from drug sensitivity screening had varying degrees of ability to secrete pyocyanin, and strains with stronger pyocyanin secretion ability were more distributed in the MDR-PA group (Figure 3C). According to the production of pyocyanin, the MDR-PA group had a stronger ability to produce pyocyanin than the S-PA group (r = 0.96, *p* < 0.05), divided into strong, medium, and weak grades (Figure 2C).

### 3.3. Gene Typing Based on Homology Analysis

**Figure 5:**
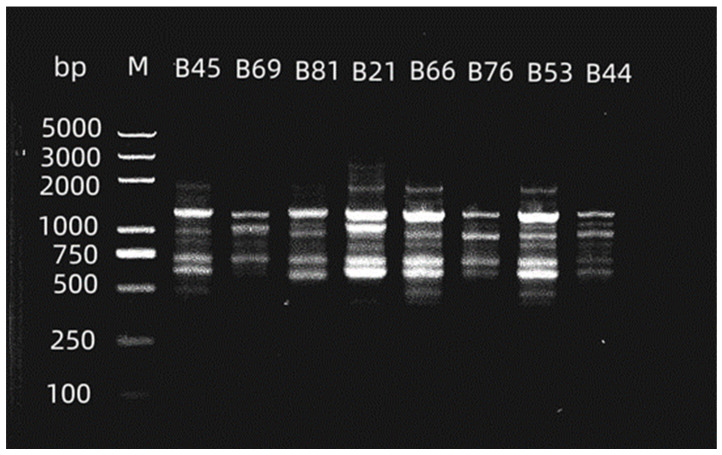
RAPD genotype mapping of the eight PA strains with the strongest expression of virulence factors (B45, B66, B53, and B21 are type A; B69 is type B; and B81, B76, and B44 are type C).

Based on the above, the eight strains with the strongest expression of virulence factors were identified and named B44, B45, B69, B53, B66, B21, B76, and B81, respectively. Homology analysis was performed to genotype the strains. The difference in the number of electrophoretic bands among eight strains of PA was within three, indicating that the strains with the strongest expression of virulence factors were closely related [14,15], suggesting a high degree of gene homology (Figure 5).

### 3.4. Correlation Matrix Analysis Between Virulence Factors

The correlation matrix between the phenotypic expression of pyocyanin and other virulence factors is listed in Table 3. The results showed a high correlation between pyocyanin and biofilm formation (*r* = 0.7408, *p* > 0.05), a moderate correlation with elastase (*r* = 0.4128, *p* < 0.0001), and a weak correlation with cluster motility (*r* = −0.1577, *p* < 0.0001). There is a moderate correlation between cluster movement and the production of elastase (*r* = 0.3257, *p* > 0.05), and a weak negative correlation with the formation of biofilms (*r* = −0.2529, *p* < 0.0001). The activity of elastase is almost uncorrelated with the presence of biofilms (*r* = 0.1033, *p* < 0.0001).

## 4. Discussion

Biofilm (BF) is an important virulence factor in the development of skin and soft tissue infections, duct and medical device related infections, dental caries, and chronic infections, and is therefore considered an important defense mechanism and pathogenic marker of MDR-PA isolates [16,17]. Hyun et al. [18] evaluated the biofilm formation of carbapenem-resistant PA isolates and found that over 92% of clinical isolates were able to form biofilms. Shahraki et al. [19] concluded that biofilm production was significantly higher in multidrug-resistant (MDR) isolates. The formation of biofilm can promote the increase of drug resistance. Karami et al. [20] found a strong positive correlation between biofilm formation and multidrug resistance status in a study involving 78 clinical isolates of PA. The research results of Eladawy et al. [21] indicate that there is no correlation between antibiotic resistance, biofilm formation, and the presence of genes encoding selected virulence factors. In this study, the amount of biofilm formation in the MDR-PA group was significantly higher than that in the S-PA group, consistent with the results of Karami et al. but different from those of Eladawy et al. This may be related to the strain source, different experimental conditions, and complex regulatory mechanisms of biofilm formation.

Pyocyanin, a blue-green water-soluble pigment with redox activity and strong toxicity, is often detected at high concentrations in the airway tissue and sputum of CF patients, leading to chronic lung infections and bronchiectasis. Márió Gajdács et al. [22] and Poonam Naik1 et al. [23] used different research methods and found that the expression of pyocyanin content was higher in the MDR group. When high concentrations of pyocyanin are detected in sputum culture, caution should be exercised in selecting antibiotics and reducing empirical use.

Elastase can degrade tissue matrix proteins and plasma proteins, causing local inflammation, while also breaking down tissue immunoglobulin, which is beneficial for the invasion and metastasis of PA and can easily lead to bacteremia [24]. Statistical analysis showed that all PA strains had the ability to form elastase. The distribution of strains with stronger elastase secretion ability was more pronounced in the S-PA group. There may be a synergistic effect between the virulence factors of PA, and the expression and function of other virulence factors may promote the secretion of elastase. In sensitive strains, this synergistic effect may be more significant, leading to an increase in LasB secretion [23]. But there was no correlation between protease activity and antibiotic sensitivity. Cluster movement was closely related to the pathogenicity of PA and could greatly enhance PA’s adaptability to the environment and resistance to various antibiotics. All clinical isolates of PA in the experiment had the ability to cluster and exhibited different phenotypes, which was consistent with the results of Ha et al [25].

With the widespread use of antibiotics, PA resistance is developing rapidly. Multi-drug-resistant infections are associated with high incidence rate, mortality, and economic costs in health care institutions worldwide. In view of this, when PA infection is diagnosed clinically, carbapenems, beta lactams, and cephalosporins should be excluded or reduced, and traditional Chinese medicine with antibacterial properties can be used to replace conventional antibiotics, which are prone to inducing resistance [26].

## 5. Conclusions

In this work, we explored the correlation analysis between the multi-drug resistance phenotype and virulence factor expression of clinical PA. The results of this study showed that MDR-PA had the strongest resistance rates to cefepime, cefazolin, ampicillin, and imipenem, reaching up to 100%. At the same time, we conducted phenotype testing on 32 PA isolates screened for drug sensitivity and found that the MDR-PA group had significantly higher biofilm formation ability and average levels of pyocyanin production than the S-PA group. However, there was no significant difference between the MDR-PA group and the S-PA group in terms of cluster motility and average secretion level of elastase. In addition, a moderate correlation was found between biofilm formation and imipenem resistance. Strains with strong expression of virulence factors have little difference in gene bands, indicating high homology. The analysis of virulence factor matrix shows that the correlation between different virulence factors is quite complex. Previous reports [27,28] have revealed that the production of pyocyanin, cluster motility, and elastase secretion levels are related to the pathogenicity of PA, and the ability to form biofilms is closely related to bacterial pathogenicity and drug resistance.

The correlation between high drug resistance and high toxicity phenotypes is controversial [29]. There is an interaction between bacterial virulence and resistance, and the associated biological costs depend on multiple factors, including the types of bacteria involved, virulence and resistance mechanisms, ecological niche, and host characteristics [30]. The specimens and methods used in different studies vary, and the phenotypes and sensitivity trends of isolates also differ, leading to significantly different conclusions. In the future, further in-depth research should be conducted to explore their relationships.

## Figures and Tables

**Table 1:** Biofilm formation of clinical isolates of PA.

Biofilm	Formation Quantity		Formation Degree	
	Possession	Nothing	Weak	Medium
Clinical bacterial strains	93.75%	6.25%	73.3%	26.7%

**Table 2:** Correlation analysis between the degree of biofilm formation and antibiotic resistance in PA.

Types of Antibiotics		CIP	PIP	AMP	CPI	GEN	CZ	NOR	IPM
Formation of biofilm	Correlation coefficient (*r=*)	n.r	n.r	n.r	n.r	n.r	n.r	n.r	0.488
	Statistical value (*p=*)	0.166	0.082	n.r	n.r	0.082	n.r	0.082	0.040

Here, “n.r” represents no correlation.

**Table 3:** Correlation matrix analysis between pyocyanine and other virulence factors in PA.

Virulence Factor	Pyocyanin (OD_520_)	Cluster Movement (cm^2^)	Elastase (mm)	Biofilm (OD_590_)	
Pyocyanin (OD_520_)		*p* < 0.0001	*p* < 0.0001	0.064052	Statistical figures (*p=*)
Cluster Movement (cm^2^)	−0.1577		0.185145	*p* < 0.0001	
Elastase (mm)	0.4128	0.3257		*p* < 0.0001	
Biofilm (OD_590_)	0.7408	−0.2529	0.1033		
Pearson correlation coefficient (*r=*)					

## Data Availability

Data is contained within the article.
